# Physical activity maintenance in colorectal cancer survivors after an exercise intervention applying behaviour change techniques: a systematic review and meta-analysis

**DOI:** 10.1007/s11764-024-01654-8

**Published:** 2024-08-05

**Authors:** Saioa Agirre-Elordui, Julen Fernández-Landa, Jurgi Olasagasti-Ibargoien, Arkaitz Castañeda-Babarro

**Affiliations:** 1 Department of Physical Activity and Sports, Faculty of Education and Sport, University of Deusto, Bilbao, Spain; 2 Department of Physical Activity and Sports, Faculty of Education and Sport, University of Deusto, Donostia, Spain

**Keywords:** Physical activity, Colorectal cancer survivors, Behaviour change techniques, Maintenance, Adherence, Long term

## Abstract

**Purpose:**

The purpose of this systematic review and meta-analysis is to determine the long-term effect of combined physical activity (PA) and behaviour change techniques (BCT) interventions in PA maintenance of colorectal cancer survivors (CRCS) and identify the most frequent BCT implemented in them.

**Methods:**

PRISMA recommendations were followed. Databases were searched for randomized controlled trials up to October 2023. Studies in which CRCS completed a PA intervention based on any Theoretical Model of Behaviour Change (TMBC) and a subsequent follow-up period were included. Between-group differences at baseline and after follow-up were pooled for meta-analysis. BCT codification was performed using the BCT taxonomy v1. Methodological quality and evidence certainty were also assessed.

**Results:**

Five studies involving 906 CRCS met the inclusion criteria. PA interventions applying BCT showed a significant change with a small positive effect (pooled SMD = 0.22 (0.09, 0.35)) on the PA after a follow-up period between 3 and 12 months. Twenty-two different BCTs were identified (mean 17.2, range 15–19) of which 12 were common across all interventions.

**Conclusions:**

PA and BCT interventions have been found to be effective in improving the long-term maintenance of PA in CRCS. Further studies with higher methodological quality are needed to confirm these findings.

**Implications for Cancer Survivors:**

Aerobic exercise, pedometers, PA diaries and educational materials seem to be important aspects to achieve sustainable adherence to an active lifestyle over time. Supervision, access to fitness areas and applying some BCT appear to be differentiating features to obtain more successful PA maintenance.

## Introduction

Cancer is a leading cause of death worldwide [1], ranking second among non-communicable diseases [2], and with growing incidence expected to increase by 50% in the next two decades [3]. Specifically, for colorectal cancer (CRC), which is the third most common cancer in the world, the incidence could increase by up to 63% between now and 2040 with 3.2 million new cases per year [4]. However, the survival rate for cancer is increasing globally due to advances in early diagnosis and treatments, so as a result, there is an expected increase in the number of colorectal cancer survivors (CRCS) in the coming years [1].

Usually, patients with CRC are treated with chemotherapy, sometimes in combination with radiotherapy and surgery [5]. These treatments can result in long-term side effects, including fatigue, which may persist for up to 5 years after completing treatment or even longer [6]. Depressive symptoms are another of the most frequent effects, along with reduced levels of physical condition and deficiencies in social and occupational activities that can lead to a significant deterioration in quality of life, which in turn, may contribute to a higher recurrence of the disease [5, 7].

Against this background, physical activity (PA) has been shown to be a safe and effective non-pharmacological and non-invasive intervention [8] with benefits at physical, psychological and social levels [9]. PA improves functional status, reduces fatigue, increases quality of life and reduces the probability of disease recurrence and mortality [5, 10, 11]. Nevertheless, approximately 50% of CRCS are not active enough [12]. Among the reasons reported by the CRCS to explain the physical inactivity, those related to the disease and its symptoms, such as fatigue, as well as, time and availability difficulties, were the most common ones [13, 14]. In the same way, socio-environmental factors, such as the lack of family support and proximity to sports facilities, were also perceived as barriers associated with lower levels of PA [15] that can lead to poor adherence to an active lifestyle in CRCS [16, 17].

In order to study PA adherence in this population, different randomized controlled trials have been conducted with favourable results for various types of interventions. On the one hand, exercise-based interventions have been found to be effective in producing PA changes in CRCS [18, 19]. On the other hand, an increasing number of studies are now implementing interventions based on theoretical models of behavioural change (TMBC) or behavioural change techniques (BCT), which have also been proven to be effective in improving PA levels in other oncology populations [20]. According to different meta-analyses, both PA [21] and BCT [22] appear to be useful in increasing PA in CRCS following an intervention period. However, it is unclear whether these improvements are sustained over time after the end of the intervention and what their long-term effects on PA behaviour are.

Although both PA and BCT interventions have been shown to be effective independently, the evidence is inconsistent when it comes from studies that apply both interventions together. In accordance with one meta-analysis, PA applying BCT can achieve an increase in PA at least 3 months after the end of the intervention period [23]. In contrast, a systematic review published recently indicated that only five out of the 21 studies included (24%) showed significant improvements in PA levels at least 6 months after the combined PA and BCT intervention [24]. Furthermore, these trials were conducted in samples with mixed cancer types, in which the number of participants for each type of cancer was uneven. In addition, the specific characteristics of each type of cancer (location, treatment, severity, prognosis) and of the population itself that is mainly affected (gender, age) could result in these interventions differing in their level of effectiveness in increasing PA in the long term. According with that, it has been observed that there are differences in perceived barriers to PA practice according to cancer type [17]. Particularly, for CRC, symptoms and specific treatments are one of the main reasons why those who survive are less likely to be physically active [13]. For these reasons, it is important to determine the effectiveness of interventions designed from a cancer-specific approach, and for CRCS, the specific effect is still unknown.

While combined PA and BCT interventions could contribute to better maintenance of PA levels, there is some controversy about their sustainability after the intervention period. Additionally, their applicability in CRCS is questionable. Therefore, it is important to identify and collect data from various studies to understand the long-term efficacy of PA and BCT, and hence, to generate knowledge about the relevance of this type of intervention to achieve sustainable PA adherence in this population. The aim of this study is to conduct a systematic review and meta-analysis (SRM) to determine the durability of the effect of combined PA and BCT interventions on long-term PA adherence in CRCS. The secondary objective of this SRM is to identify the most frequent BCT and analyse their application in the most effective interventions.

## Material and methods

The SRM was conducted following the recommendations of the Preferred Reporting Items for Systematic Reviews and Meta-analysis (PRISMA) 2020 statement [25], and the protocol was registered in the International Prospective Register of Systematic Review (PROSPERO): CRD42024492832.

### Literature searching strategies

The search for relevant publications was replicated across three electronic databases (PubMed, Web of Science and Scopus) until October 2023. The search strategy used in each database followed the PICOS principle (population, intervention, comparison, outcome and study design), and both indexed and free words were combined with Boolean operators to conduct literature retrieval: ((“colorectal neoplasms”[Mesh]) OR (“colorectal neoplasm”) OR (“colorectal cancer”)) AND (“survivors”) AND ((“exercise”[Mesh]) OR (“exercise”) OR (“physical activity”) OR (“exercise intervention”) OR (“physical activity intervention”)) AND ((“adherence”) OR (“maintenance”) OR (“efficacy”) OR (“physical activity level”) OR (“physical activity change”) OR (“behavior change”) OR (“behaviour change”)).

The literature searching, studies selection and data extraction were carried out independently by two authors (SA and AC), and disagreements were solved by consensus or involving a third author (JF) when it was needed. All quality assessments and BCT codification were also replicated by two authors (SA and AC), and a third author (JO) was involved to clarify possible discrepancies.

### Eligibility criteria

The criteria used to determine the inclusion and exclusion of studies were also based on the PICOS question.

i) Population: clinically confirmed CRCS older than 18 years. CRCS was considered as those survivors who had completed their primary treatment for CRC and were in a control or revision period
ii) Intervention: any type of intervention aimed at PA that also included a follow-up period after the intervention. Interventions with specific goals for PA practice were included irrespective of whether they were received through exercise sessions, behavioural counselling, supervised or unsupervised activities and regardless of the intensity, volume or frequency. Similarly, the follow-up period was not limited to any specific duration to be included. The studies were also required to report the implementation of any TMBC in addition to the PA intervention
iii) Comparison: any control group that did not include PA practice. Could include usual care interventions or educational materials.
iv) Outcomes: PA at the baseline and after the follow-up period
v) Study design: randomized controlled trials

Studies were excluded if they did not meet all the above criteria and if they were published in a language other than English.

### Studies selection and data extraction

Studies selection was carried out by examining the title and abstract first and by full-text assessment to determine eligibility then. The selection of potentially eligible studies and data extraction were recorded in an Excel file. The data collected included the following: publication data (journal, publication year, author(s) and title), study design, sample size, mean age of participants, duration of the intervention and follow-up, description of the intervention and control conditions, measurement tool for the PA and the TMBC employed. The possible outcome measures for PA in this SRM were as follows: weekly minutes or times of moderate-vigorous physical activity (MVPA) and weekly metabolic equivalent task (MET) per hour. If additional research information was required, the supplementary online information was accessed, or, when it was needed, the corresponding author was contacted by email to obtain additional data.

### *Meta*-analysis

A meta-analysis was performed using the “metafor” package of R software (R Foundation for Statistical Computing, Vienna, Austria). Furthermore, for those cases in which more than one outcome measure per study was included to evaluate PA changes, the “MAd” package was employed, which reduces errors that affect the weight that the meta-analysis attributes to each study [26]. Therefore, a single estimation of the effect size was calculated [27] applying a within-study correlation of 0.7 [28]. Baseline and follow-up means in PA were used to obtain a weighted estimation of standardized mean difference (SMD) as Hedges’G [29] and were classified as trivial (< 0.2), small (0.2–0.3), moderate (0.4–0.8) or large (> 0.8) according with Cohen’s criteria [30]. Following the DerSimonian and Laird method [31], the variance was calculated with the inverse variance random-effects model and with a 0.7 correlation coefficient [32]. Statistical significance was set at *p* < 0.05 with a 95% confidence interval (95% CI; (lower bound, upper bound)).

The heterogeneity assessment was based on the restricted maximum-likelihood estimator of tau-square, and the inconsistency between the studies included in the SRM was evaluated with *I*^2^ statistic. The inconsistency was considered small when *I*^2^ was less than 25%, medium when it was between 25 and 50% and large when it was greater than 50% [33].

The potential publication bias was visually evaluated in the funnel plot asymmetry and also was assessed by Egger’s regression test [34]. In the same way, to identify the missing studies on either side of the funnel plot, Duval and Tweedie’s trim and fill method was used [35].

### Behaviour change techniques codification and analysis

Following the “BCT taxonomy v1”, the BCT applied in the intervention group (IG) of each study were identified. The BCT taxonomy was validated to codify and classify in a standardized way 93 different techniques for health behaviour change [36]. The target behaviour of the application of these BCT was PA. The techniques were coded as informed or not informed, and a synthesis of the most and least frequent techniques was subsequently carried out. The total number of BCT applied in each intervention was also summarized [37].

In addition, following the results of the meta-analysis, studies were classified as very promising (significant effect in favour of intervention), promising (non-significant effect in favour of intervention) or not promising (no effect in favour of intervention) in order to identify and describe which techniques could differentiate between successful and unsuccessful interventions [38].

### Risk of *bias* and evidence certainty assessments

Methodological quality and risk of bias were evaluated using the standardized Cochrane Collaboration’s bias risk tool [39]. The assessment criteria rated as “low risk”, “some concerns” or “high risk” were employed in five different aspects of individual studies: randomization process, intervention blinding, missing outcome data, measurement of the outcome and selection of the reported results.

On the other hand, to assess the certainty of the evidence, the system proposed by the Grading of Recommendations Assessment, Development and Evaluation (GRADE) guide was followed [40]. The grading of PA outcome was assessed in terms of study design, risk of bias, imprecision, inconsistency and indirectness. Following these criteria, the evidence was considered as high, moderate, low or very low certainty.

## Results

### Literature searching

A total of 525 studies were initially identified for the SRM. An additional study was detected after reviewing the bibliographic references of different reports. Subsequently, 207 studies were excluded as duplicates, and 319 articles were screened by title and abstract, 280 of which did not meet the inclusion criteria and were removed. As a result, 39 studies remained for review in full-text.

**Fig. 1:**
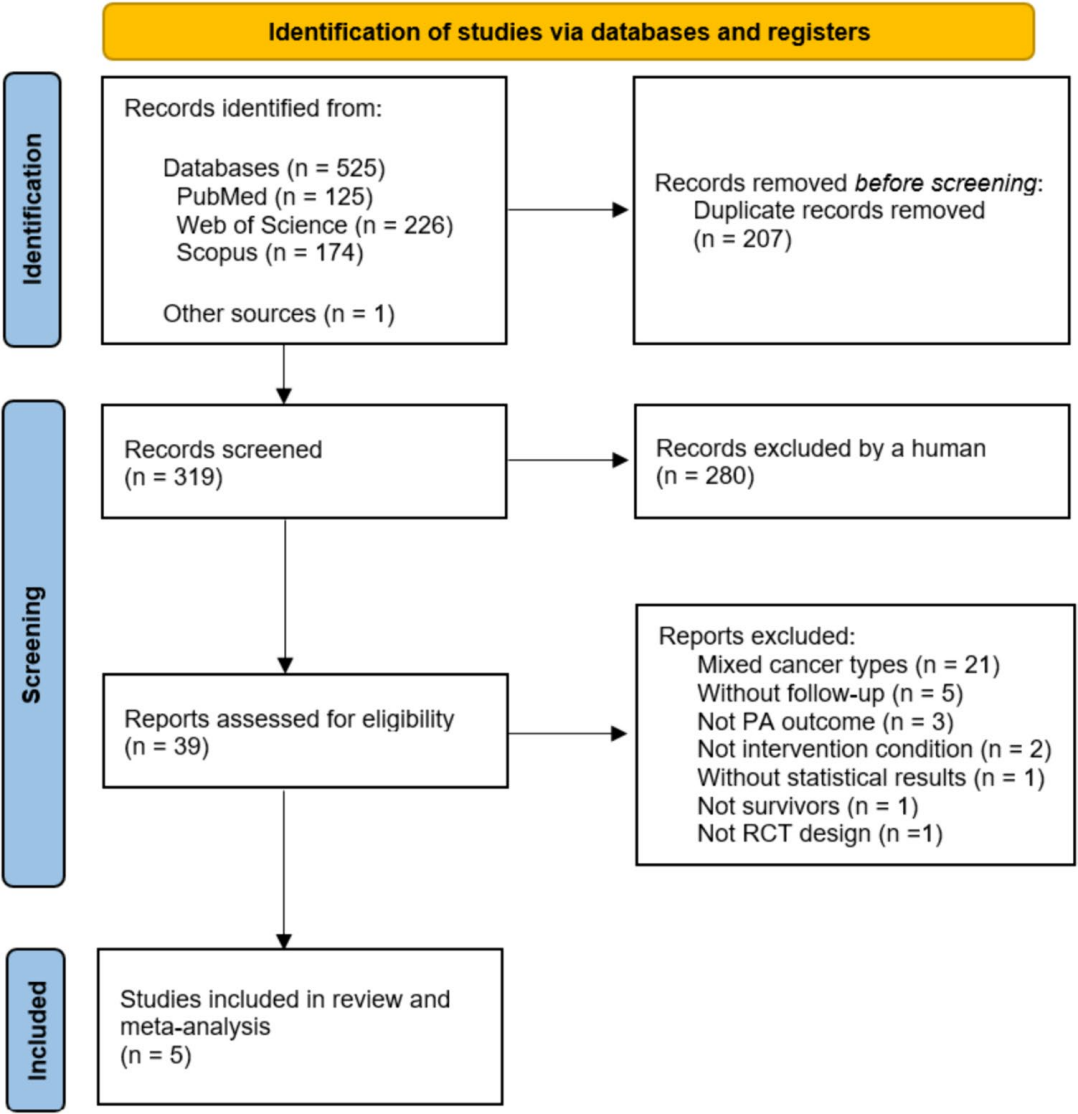
Flow diagram of the selection process of included studies

After full-text review, 21 articles were excluded because they reported combined data for different types of cancer without providing information on individual outcomes for CRC. In addition, five articles were excluded because they did not include a follow-up period after the intervention, other three because of outcome inconsistencies, two articles did not meet the intervention conditions, one study was conducted with patients still on treatment and not with survivors, one study did not provide statistical results because of a small sample size, and one study did not follow the study design specified in the inclusion criteria. Finally, the SRM included five studies (Fig. 1).

### Demographic characteristics of the included studies

The main characteristics of the studies are shown in Table 1. The five studies finally included in the SRM involved a total of 906 CRCS. The range of participants varied between 42 and 347, with mean ages ranging from 55.6 to 67.8 years old. Males were slightly more prevalent than females in three of the studies [41–43], whereas the female sex was slightly more prevalent in the other two [44, 45]. However, both sexes were equally represented in all five studies, with each comprising around 50% participation.

**Table 1:** Main characteristics and results of the included studies

Authors and year	Number of participants	Country	Mean age (SD), % sex	Measuring tool	Outcome measures	PA mean (SD) at baseline	PA mean (SD) post-intervention	PA mean (SD) post-follow-up	Results*
Courneya et al. (2016) [41]	IG 106; CG 105	Canada; Australia	60, 46% M	TPAQ	Min/w MVPA	IG 16.5 (22.4); CG 16.6 (19.2)	(n.i)	IG 31.2 (30.7); CG 21.7 (20.2)	PI (n.i); ↑ at 6 months PF
Hawkes et al. (2013) [42]	IG 171; CG 176	Australia	IG 67.8 (9.2), 56.1% M; CG 64.9 (10.8), 51.7% M	GLTPAQ	MET-hour/w MVPA	IG 58.9 (132.9); CG 52 (112.5)	IG 85.1 (197.9); CG 66.7 (139.2)	IG 85.2 (181); CG 54.3 (120)	↔ PI; ↑ at 6 months PF
Lee et al. (2018) [43]	IG 51; CG 48	China	65.2 (10.1), 63.2% M	ActiGraph accelerometer	Min/w MVPA	IG 461.8 (239.6); CG 473.3 (267.3)	IG 570.1 (204.2); CG 616.6 (310)	IG 631.8 (281.4) at 6 months, IG 680.5 (259.8) at 12 months; CG 676.2 (367.3) at 6 months, CG 642.4 (294.7) at 12 months	PI (n.i); ↔ at 6 months PF; ↔ at 12 months PF
Mayer et al. (2018) [44]	IG 105; CG 102	USA	IG 59.34 (13.7), 49% M; CG 57.84 (14.5), 48% M	GLTPAQ	Times/w MVPA	IG 14.3 (13.7); CG 13.16 (12.9)	IG 31.42 (23.12); CG 25.51 (20.54)	IG 29.8 (27); CG 25.89 (23.99)	↑ PI; ↔ at 3 months PF
Pinto et al. (2013) [45]	IG 19; CG 23	USA	IG 59.5 (11.2), 40% M; CG 55.6 (8.24), 46% M	7D-PAR	Min/w MVPA	IG 37.6 (72.49); CG 28.65 (31.51)	IG 214 (n.i); CG 97 (n.i)	IG 172.11 (172.2) at 3 months, IG 148.63 (209.82) at 6 months; CG 85.23 (126.44) at 3 months, CG 86.54 (103.44) at 6 months	↑ PI; ↔ at 3 months PF; ↔ at 6 months PF

*7D-PAR* 7-Days Physical Activity Recall, *CG* control group, *GLTPAQ* Godin Leisure-Time Physical Activity Questionnaire, *IG* intervention group, *n.i* not informed, *M* male, *MET* metabolic equivalent of task, *MVPA* moderate-vigorous physical activity, *PA* physical activity, *PF* post-follow-up, *PI* post-intervention, *SD* standardized deviation, *TPAQ* Total Physical Activity Questionnaire, *USA* United States of America

^*^Between-group differences from baseline

↑Significant increases in PA between groups

↔ Non-significant increases in PA between groups

### Outcome measures

Regarding the assessment of PA (Table 1), both objective measurement tools, such as ActiGraph accelerometer [43], and subjective or self-reported tools, such as the “Godin Leisure-Time Physical Activity Questionnaire (GLTPAQ)” [42, 44], the “Total Physical Activity Questionnaire (TPAQ)” [41] or the “7-Days Physical Activity Recall (7D-PAR)” [45], were used. As defined in the inclusion criteria, outcome measures were reported in minutes [42, 43, 45] and times [44] per week of MVPA and in MET-hour per week [41].

### Intervention effect between groups

The results about the effect of the interventions on PA across different timepoints are shown in Table 1. All studies included in the SRM reported improvements in PA levels from baseline to post-follow-up; however, only two of them found statistically significant differences between groups with increases in MVPA of 23.7 min/week (*p* = 0.007) [42] and 10 MET-h/week (*p* = 0.02) [41] over the control group (CG).

In addition, four of the included studies also reported on post-intervention PA outcomes, although only three of them performed statistical analysis to study between-group differences [42, 44, 45]. From baseline to post-intervention, PA increased in all three studies, even though the changes were only statistically significant in two of them with between-group differences of 5.91 times/w (*p* = 0.045) [44] and 117 min/w (*p* = 0.021) [45] of MVPA in favour of IG. In contrast, Hawkes et al. [42] did not find between-group statistically significant differences from baseline to post-intervention (*p* = 0.172). In the case of Lee et al. [43], despite the fact that an increase in PA levels post-intervention was also observed, its effectiveness was not statistically analysed.

Regarding changes between post-intervention and post-follow-up, all studies that informed on these measures observed a decrease in CG PA levels [42, 45], with the exception of one that increased [43], and another that was unchanged [44]. Similarly, in all but one IG [43], PA levels decreased from post-intervention to post-follow-up [44, 45], and in another, they were maintained [42]. However, IG PA levels remained higher than CG levels after the follow-up, although three of the studies did not report whether that between-group difference was statistically significant [42–44], and in the other, it was not (*p* = 0.149 at 3 months post-follow-up timepoint and *p* = 0.223 at 6 months post-follow-up timepoint) [45].

### *Meta*-analysis

**Fig. 2:**
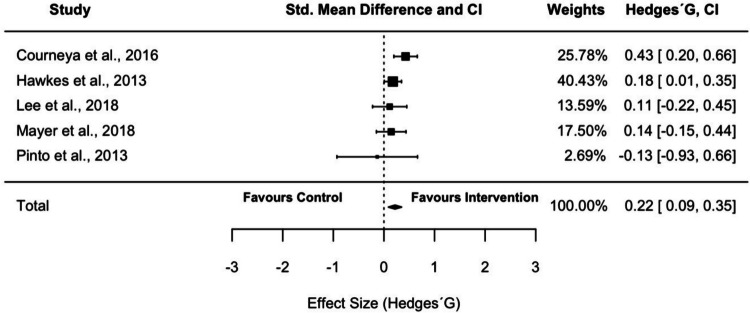
Forest plot of included studies

**Fig. 3:**
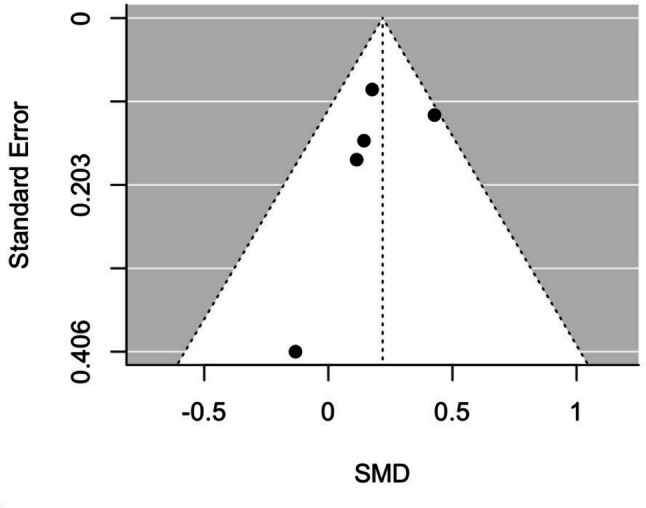
Funnel plot of the included studies

Following meta-analysis results (Fig. 2), PA interventions applying BCT showed a significant (*p* < 0.01) and small (pooled SMD = 0.22 (0.09, 0.35)) effect on the PA. Furthermore, the heterogeneity analysis indicated a small inconsistency (*I*^2^ 15.4%; *p* = 0.32). After observing the funnel plot asymmetry (Fig. 3) and performing Egger’s regression test (df = 3, *p* = 0.52), no possible publication bias was identified. The Duval and Tweedie trim and fill test suggested that two studies are missing on the right side of the plot.

### Intervention and control conditions

The main characteristics of the intervention and control conditions are detailed in Table 2. PA and BCT interventions comprised a duration between 3 [45] and 12 months [43] although most were carried out over a 6-month period [41, 42, 44]. In the same way, the length of follow-up periods also ranged from 3 [44, 45] to 12 months [43]. Furthermore, two studies had more than one follow-up period after the end of the intervention: at 3 and 6 months [45] and at 6 and 12 months [43].

**Table 2:** Intervention and control characteristics of included studies

Authors and year	Intervention and follow-up length	Intervention condition	Intervention goal	Theoretical Model of Behaviour Change	Control condition
Courneya et al. (2016) [41]	6 months I 6 months F	- 12 supervised exercise sessions - Access facilities to fitness area - Pedometer and PA diaries to register PA - Frequent and ongoing contact including some face-to-face sessions with qualified staff - Educational written materials like an exercise guidebook developed specifically for CRCS - BCT based on a validated theoretical model	AER at least 10 MET-h/w and sustain this change	Theory of Planned Behaviour	General health educational materials promoting PA and healthy nutrition as standard surveillance follow-up
Hawkes et al. (2013) [42]	6 months I 6 months F	- 11 telephone-delivered HC sessions that include cancer experience and symptoms, lifestyle behaviours and strategies to improve HBC - Pedometer and PA diaries to register PA - Motivational postcard to promote HBC - Educational written materials and study´s newsletter, same as CG	10,000 steps/d	Acceptance Commitment Therapy	Four freely available educational brochures on understanding CRC and cutting cancer risk, diet and PA Quarterly study’s newsletter to enhance participant retention
Lee et al. (2018) [43]	12 months I 6 and 12 months F	- Face-to-face motivational interviews - Pedometer and PA diaries to register PA - Motivational phone calls - Mailed booklets and newsletters about stage-of-change matched - Group meetings	60 min of MVPA across 5 d/w	Theory of Planned Behaviour and Health Action Process Approach	5 mailed booklets about general health advice that encouraged healthy lifestyles by eating a wide variety of food, more fruit and vegetables, increasing PA levels, quitting smoking and avoiding alcohol abuse
Mayer et al. (2018) [44]	6 months I 3 months F	- Pedometer to register PA - Educational materials about life after cancer, same as CG -Motivational messages, answers to fitness questions and exercise videos -Smartphones with the *SurvivorCHESS* application: • *My Tracket*, PA logging and goal setting • *My Friends*, to provide social support by individual messaging and discussion forum • *Be Mobile*, information modules on PA and health • *My Cancer Care*, tools for adapting to life after cancer diagnosis	150 min/w of daily activities	Self-Determination Theory	Pedometer and educational materials about life after cancer
Pinto et al. (2013) [45]	3 months I 3 and 6 months F	- Pedometer and PA diaries to register PA -Weekly calls to monitor PA participation, identify health problems, solve barriers to PA and reinforce participants for their efforts - Individual feedback about PA - Letter with participants’ progress - CRC survivorship tip sheet, same as CG	10 min × 2 d/w increasing gradually to 30 min × 5 d/w (AER at 64–76% of HRmax.)	Transtheoretical Model of Behaviour Change	Weekly calls and CRC survivorship tip sheets to maintain participants’ retention

*AER* aerobic activities, *BCT* behaviour change techniques, *CG* control group, *CRC* colorectal cancer, *d* days, *F* follow-up length, *h* hours, *HBC* health behaviour change, *HC* health coaching, *HRmax.* maximum heart rate, *I* intervention length, *MET* metabolic equivalent task, *min* minutes, *MVPA* moderate-to-vigorous physical activity, *PA* physical activity, *w* week

The intervention goals were primarily to increase the duration and frequency of aerobic PA and to sustain that change over time. To this purpose, participants were provided with pedometers, PA diaries and educational materials on healthy lifestyle habits and cancer [41–45]. Moreover, one of the studies established an intervention of 12 supervised exercise sessions in which access to fitness spaces was also facilitated [41]. Additionally, the interventions included motivational face-to-face interviews [43], messages [44] or written materials [42], telephone consultations [42, 43, 45] and exercise videos [44]. One of the studies created a smartphone application that provided all these resources across different PA promotion and registration services, social support and tools for adjusting to life after cancer [44]. In terms of the TMBC followed in the design of each intervention, five different theoretical models were used: the Theory of Planned Behaviour [41], which in one instance was applied together with the Health Action Process Approach [43]; the Acceptance Commitment Therapy [42]; the Self-Determination Theory [44] and the Transtheoretical Model of Behaviour Change [45].

The control conditions of all studies were characterized by the provision of general health and cancer education materials, although one study also provided pedometers [44], and another conducted weekly calls to maintain participant retention [45].

### Behaviour change techniques codification and analysis

The BCT identified are listed in Table 3. The interventions included at least 15 [44] of the possible 93 techniques and a maximum of 19 [41] (mean = 17.2 BCT). Considering the set of interventions, 22 different BCT were applied, of which 12 were common: *1.1. goal setting (behaviour), 1.3. goal setting (outcome)*, *1.4. action planning*, *1.5. review of behavioural goal(s), 2.2. feedback on behaviour, 2.3. self-monitoring of behaviour, 4.1. instructions on how to perform the behaviour, 5.1. information about health consequences, 7.1. prompts/cues, 8.1. practice/rehearsal of the behaviour, 9.1. credible source, 12.5. adding objects to the environment* and *15.1. verbal persuasion about ability.*

**Table 3:** Behaviour change techniques codification of the included studies

	Courneya et al. (2016)	Hawkes et al. (2013)	Lee et al. (2018)	Mayer et al. (2018)	Pinto et al. (2013)	Total (studies)
1.1. Goal setting (behaviour)	Behavioural support sessions	Telephone HC sessions	Face-to-face motivational interviews	Smartphone with *SurvivorCHESS* application, *My Tracket* service	Telephone counselling	5
1.2. Problem solving	Behavioural support sessions	Telephone HC sessions	-	-	Telephone counselling	3
1.3. Goal setting (outcome)	Behavioural support sessions	Telephone HC sessions	Face-to-face motivational interviews	Smartphone with *SurvivorCHESS* application, *My Tracket* service	Telephone counselling	5
1.4. Action planning	“Planning Worksheet” with specifics on who, what, when, where and how	Telephone HC sessions	Face-to-face motivational interviews	Smartphone with *SurvivorCHESS* application, *My Tracket* and *Be Mobile* services	Telephone counselling	5
1.5. Review behaviour goal(s)	Behavioural support sessions	Telephone HC sessions	Motivational phone calls	Smartphone with *SurvivorCHESS* application, *My Tracket* service	Individual feedback throughout telephone counselling Progress-letter	5
2.2. Feedback on behaviour	Pedometer Supervised exercise sessions	Pedometer	Pedometer	Smartphone with *SurvivorCHESS* application Pedometer	Pedometer	5
2.3. Self-monitoring of behaviour	PA registration diaries	PA registration diaries	PA registration diaries	PA registration diaries throughout *SurvivorCHESS* application	PA registration diaries	5
3.2. Social support (practical)	-	-	Group meetings	Smartphone with *SurvivorCHESS* application, *My Friends* service	-	2
3.3. Social support (emotional)	Asked to bring their main PA support person to some sessions	Motivational interviewing during telephone HC sessions	Face-to-face motivational interviews	-	Motivational interviewing during telephone counselling	4
4.1. Instructions on how to perform the behaviour	Behavioural support sessions Supervised exercise sessions	Telephone HC sessions	Face-to-face motivational interviews Motivational phone calls	Smartphone with *SurvivorCHESS* application, *Be Mobile* service Online contact with PA professional	Telephone counselling	5
5.1. Information about health consequences	Behavioural support sessions and educational written materials	Educational written materials	Mailed booklets and newsletters	Smartphone with *SurvivorCHESS* application, *My Cancer Care* service Educational materials	Telephone counselling	5
5.3. Information about social and environmental consequences	-	-	Face-to-face motivational interviews	-	-	1
6.1. Demonstration of the behaviour	Supervised exercise sessions	-	Group meetings	Exercise videos	-	3
7.1. Prompts/cues	Behavioural support sessions	Motivational postcards prompt	Mailed booklets and newsletters	Motivational messages	Telephone counselling	5
8.1. Behavioural practice/rehearsal	Behavioural support sessions	Telephone HC sessions	Face-to-face motivational interviews	Motivational messages	Telephone counselling	5
8.7. Graded tasks	Behavioural support sessions	-	Face-to-face interviews and motivational phone calls	-	Telephone counselling	3
9.1. Credible source	PA professional	Health professional trained in PA	Health professional	PA professional	Professional trained on PA and behaviour change	5
9.2. Pros and cons	Behavioural support sessions	Telephone HC sessions	-	-	Telephone counselling	3
12.1. Restructuring the physical environment	Explore PA opportunities to create a supportive physical environment Access facilities to fitness areas	-	-	-	-	1
12.5. Adding objects to the environment	Pedometers and PA diaries Supervision for exercise sessions	Pedometers and PA diaries	Pedometers and PA diaries	Pedometers and PA diaries Smartphones with *SurvivorCHESS* application	Pedometers and PA diaries	5
13.2. Framing/reframing	-	-	-	-	Telephone counselling	1
15.1. Verbal persuasion about capability	Behavioural support sessions	Telephone HC sessions	Motivational phone-calls	Motivational messages	Telephone counselling	5
Total (BCT)	19	16	18	15	18	-
Meta-analysis results (SMD)	0.43	0.18	0.11	0.14	− 0.13	-
Classification	Very promising		Promising		Not promising	-

*BCT* behaviour change techniques, *HC* health coaching, *PA* physical activity, *SMD* standardized mean difference

Contrasting the studies according to the effect of the interventions, it was observed that the techniques *6.1. demonstration of the behaviour* and *12.1. restructuring the physical environment* were used only in very promising [41] or promising [43, 44] interventions and not in studies classified as not promising. In contrast, the *13.2. framing/reframing* technique was exclusively implemented in studies grouped as not promising [45]. It should be noted that techniques number *12.1* (very promising) and *13.2* (non-promising) were only used once, in the same way that technique *5.3. information about social and environmental consequences*, that was only reported once and in a study with non-significant effects in favour of the intervention [43].

### Risk of *bias* and evidence quality assessments

**Fig. 4:**
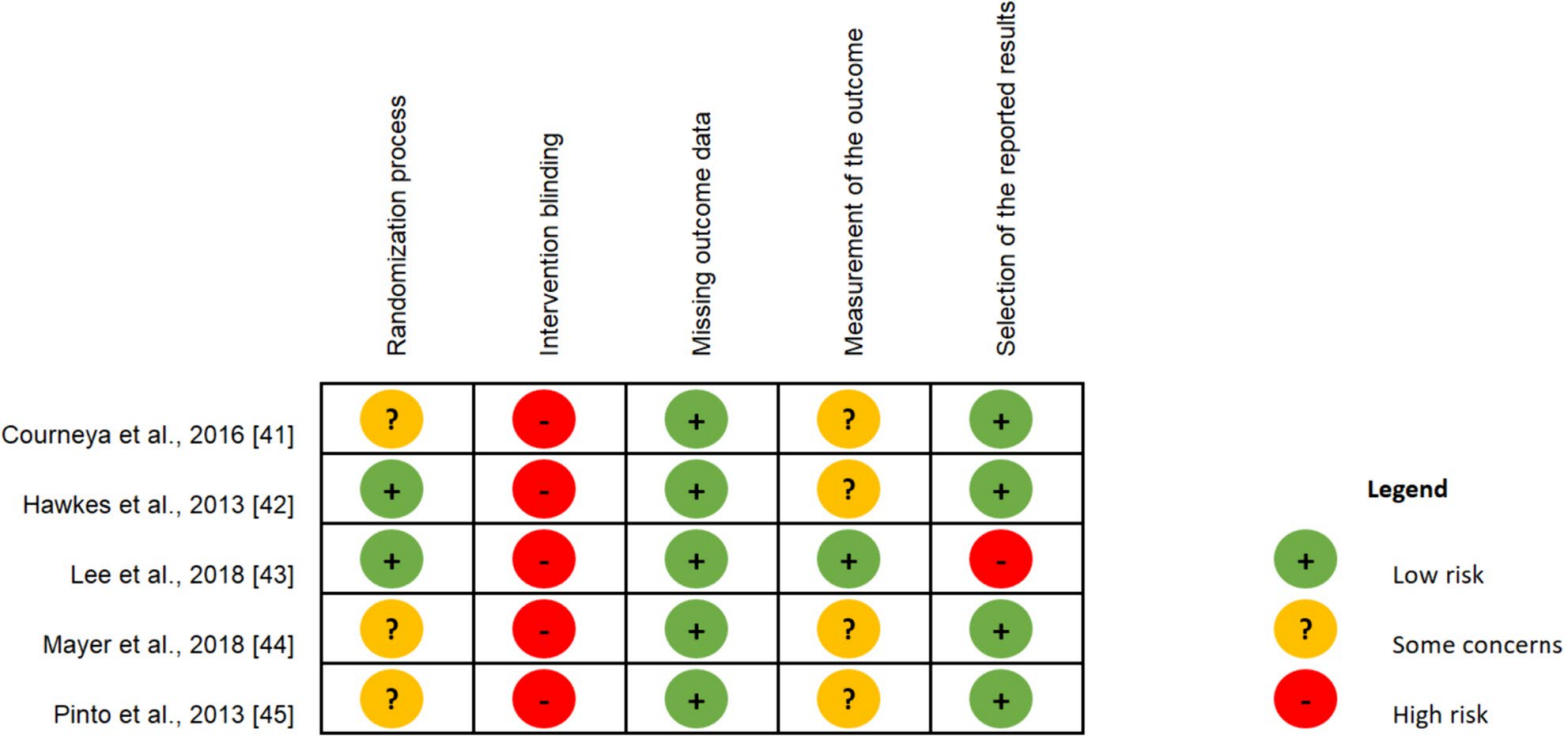
Cochrane collaboration´s risk of bias assessment of included studies

The results of the risk of bias assessment are shown in Fig. 4. The evaluation of “some concerns” with respect to randomization process derived from the fact that three studies only reported that the allocation was randomized, but did not describe the procedure used or whether it was done in a blinded way [41, 44, 45]. While the majority of studies suggested that the outcome measures were conducted by staff member who was blinded to group allocation, most measures were self-reported and therefore susceptible to potential desirability bias [41, 42, 44, 45]. Concerning blinding of the intervention to participants and instructors was assessed as “high risk” because of the challenge in enforcing blinding given the nature of the interventions. In addition, the selection of reported findings criterion was rated “high risk”, as it could lead to insufficient or inappropriate interpretation of the results, even if the description of the methodological section for the analysis was followed [43].

Regarding the quality of the evidence (Table 4), despite starting from “high quality” due to the randomized controlled trial design of the included studies, the PA outcome was downgraded one level to “moderate quality” because of the serious risk bias previously described.

**Table 4:** Evidence quality assessment following GRADE guidelines

Studies (design)	Risk of bias	Inconsistency	Indirectness	Imprecision	Publication bias	Overall sample size	Effect SMD (95% CI)	Certainty
Five (RCT)	Serious^a^	Not serious	Not serious	Not serious	Undetected	906	0.22 (0.09, 0.35)	⊕ ⊕ ⊕ ◯ Moderate

^a^Lack of blinding and some concerns for allocation and measurement of outcome

*CI* confidence interval, *RCT* randomized controlled trial, *SMD* standardized mean difference

## Discussion

The primary aim of this SRM was to investigate the long-term impact of PA and BCT interventions in PA maintenance of CRCS. Five randomized controlled trials with a total of 906 CRCS met the criteria for inclusion in the SRM, where a significant change with a small positive effect (SDM = 0.22, (0.09, 0.35); *I*^2^ = 15.4%) was found after completing a PA intervention combined with the use of different BCT and a follow-up period ranging from 3 to 12 months. This finding was consistent with previous meta-analyses conducted on both healthy inactive individuals [46] and cancer patients [20] or survivors [23] who completed PA and BCT interventions and a subsequent follow-up of at least 3 months with similar SMD not higher than 0.26.

Small effect sizes are common in these types of studies because improvements in PA can be observed not only between-groups, but also within the IG and, even more, within the CG. Grimmet et al. [23] conducted a meta-analysis to test the effectiveness of these interventions in different types of cancer (most of them in breast cancer), with follow-up periods starting at 3 months. The results showed significant increases in PA in both the IG (SMD = 0.49, 95% CI (0.32, 0.66)) and the CG (SMD = 0.21, 95% CI (0.08, 0.35)), as well as between-groups (SMD = 0.25, 95% CI (0.16, 0.35)). These results suggest that the effect of interventions on PA behaviour may be underestimated. On several occasions, it has been defined as “contamination” and is commonly found in randomized controlled trials of PA and dietary behaviour change [47], since participation in the study could be influenced by a highly biased behaviour change, irrespective of group allocation. Therefore, pooling these data in a meta-analysis may magnify type II errors, leading to an intervention being incorrectly considered ineffective because important changes in CG are ignored [48]. A possible solution to address this problem in future studies may be to offer the CG the possibility of performing the intervention after the study is completed. In this way, it may be possible to avoid the early predisposition of the CG to change their PA behaviour and comply with the indication not to change their usual PA level during the study. In addition, a larger sample size could also help minimize these type II errors.

This SRM also revealed very similar results to those presented by Mbous et al. [21] indicating the potential effectiveness of such interventions in CRCS. However, it did not consider the specific long-term effectiveness of these interventions, as studies without a subsequent follow-up period were also included. In contrast, our SRM suggested that PA changes in CRCS could be sustained for at least 3 months after the intervention and up to 12 months thereafter.

When the included trials were examined in more detail through descriptive analysis, although four studies also reported post-intervention results indicating higher levels of PA compared with those obtained at baseline, these increases were only statistically significant in two of them [44, 45]. By contrast, these same interventions were not effective during post-follow-up. Thus, no intervention that was effective post-intervention was effective post-follow-up. On the other side, there was one intervention that was not effective to increase PA significantly post-intervention but it was after the follow-up period [42]. This could be explained, on the one hand, by the contamination during the intervention period, and on the other hand, by the greater durability of the effect in the IG subjects. The fact that both groups increased PA levels during the intervention could have contributed to the non-statistically significant differences between groups. However, during the follow-up period, between-group differences became significant possibly because the IG maintained their increased levels while the CG decreased to almost their initial values.

Following on from the above, an indicator of maintenance could be that the level of PA did not decrease significantly from the post-intervention to the post-follow-up period in the IG. This implies that, while significant differences between-groups are to be expected, it is also important to consider what happens within each group. This fact reflects one of the limitations found in the scientific literature to identify the most effective interventions for the maintenance of PA. Therefore, it is necessary to consider those interventions that manage to maintain the increase in PA achieved during the intervention in the IG, without necessarily continuing to increase during follow-up, as successful.

Regarding measurement tools, despite the fact that self-reported PA is a valid and widely used tool to assess changes in PA with acceptable correlations to accelerometer measurement in CRCS [49, 50], the results should be interpreted with caution. In all but one of the studies included in the SRM [43], changes in PA were reported using self-report questionnaires. This could lead to an assumption of social desirability bias, which could overestimate the effectiveness of the intervention [21, 51]. Conversely, some studies have also identified an underestimation of PA measured by self-reported questionnaires compared to that indicated by an accelerometer [52]. Specifically, this was observed in one of the studies included in this SRM, where the GLTPAQ was used to assess participant eligibility and resulted in substantially lower PA levels compared to those obtained at baseline using accelerometer measurements [43]. It has been argued that the high walkability of the intervention environment could have contributed.

### Intervention and control conditions

Among the intervention conditions of the included studies, four common features were identified: aerobic activity, use of pedometers, PA diaries and educational materials about healthy habits and cancer. In contrast, the control conditions were characterized by the use of the same educational materials, whereas, in two of the included studies, they also provided pedometers [44] and made calls [45]. These are important aspects to consider as neither of these two studies found statistically significant differences between groups, but CG significantly increased their PA levels during the intervention. This point may suggest that providing pedometers or making calls to maintain participant retention could be enough features to produce increases in PA levels of CRCS immediately after the end of the intervention period, but insufficient to maintain or increase PA in the long term.

Regarding the intervention characteristics, it is important to mention that the inclusion criteria for this SRM were not limited to aerobic activities alone. However, previous reviews have not studied strength training as a potential intervention to increase PA [1, 5, 53]. As a result, it is unclear whether strength training can be useful as a strategy to improve adherence to PA. Insufficient adherence rates to strength programmes may be a reason why it is not commonly used as an intervention to improve PA behaviour, let alone expected to do so in the long term. A recent study implemented strength training in frail CRC patients over 70 years old and found a low adherence rate to the programme [54]. In addition, as previous studies have shown, there are difficulties in monitoring strength training in a way that reflects changes in PA levels [24]. Therefore, studies are necessary to determine the effectiveness of this type of training in changing PA behaviour and maintaining the changes over time. Nevertheless, evidence suggests that combining both types of activities could be an effective intervention that improves fatigue, depression, health-related physical fitness, body composition, quality of life and survival in CRCS [5, 55, 56], as well as producing short-term positive changes in PA levels [18, 57].

Supervision is another important component of the interventions. It is worth noting that the scientific literature reports higher adherence percentages in supervised activities [5]. However, only one of the studies in this SRM carried out the exercise in a supervised way [41], which resulted in the largest effect size for increasing levels of PA post-follow-up. Furthermore, it differed from the other interventions by facilitating access to fitness areas. This result confirms previous studies on the effectiveness of supervised exercise interventions in increasing PA in CRC patients [58]. Although other studies included in this SRM also maintained contact with participants through phone calls [42, 43, 45], text messages [44] or face-to-face meetings [43], it is unclear whether these methods alone are sufficient. A recent review suggested that supervision during the intervention could be necessary to sustain PA changes in the long term, but it could not be sufficient on its own [24]. One reason for this could be the implementation of additional BCT that supervised exercise entails. In accordance with that review and following BCT results of this SRM, some of the additional techniques that could be involved were as follows: *instructions on how to perform the behaviour*, *demonstration of the behaviour* or instantly applied *feedback on behaviour.* Furthermore, supervision could even include the use of other BCT that were not explicitly mentioned in the methodology of the studies but could be applied indirectly, for instance, *social support, information about consequences, positive reinforcement as a form of reward* or *verbal persuasion about capability.*

### Behaviour change techniques

As a secondary objective of the SRM, the aim was to identify the BCT applied in the interventions of the included studies. On average, 17.2 BCT were detected, which is higher than the average found in other reviews, between 7.6 [24] and 10.3 BCT [23]. Studies with lower means obtained wider ranges (2–13 BCT [23], 2–20 BCT [24]) than those found in this SRM (15–19 BCT) with a higher mean. The reason could be that this SRM only included studies that incorporated the use of some TMBC for the design of their interventions, resulting in a higher number of implemented BCT. Our finding was consistent with the review by Avery et al. [59] who suggested that interventions supported by TMBC and using more than 10 BCT could positively affect PA behaviour.

Although the interventions were designed on the basis of 5 different TMBC, 12 common BCT were found, of which 8 coincide with techniques reported more frequently in other studies [20, 21, 23, 24]: *goal setting, action planning, review of behavioural goal(s), feedback on behaviour, instructions on how to perform the behaviour, information about health consequences, practice/rehearsal of the behaviour* and *self-monitoring*. In contrast, the same studies mentioned that other techniques such as *problem-solving, social support, graded tasks* and *behaviour modelling* were also frequently used. While the latter BCT were not common in all studies of this SRM, they are detected in at least one of the studies classified as “very promising”, with the exception of *behaviour modelling*, which was not applied in any of them.

It should be noted that the differential techniques *demonstration of behaviour* and *restructuring the physical environment* found between the “very promising” or “promising” and “not promising” studies did not coincide with those reported in the literature, which corresponded to *action planning, social support* and *graded task* [21, 23]. However, this result could have been highly influenced by the small number of studies included in this SRM. Furthermore, the discovery of two BCT that were not typically found in previous studies suggests a potential area for future research into the applicability of these techniques, which could be previously unknown but could have influenced our results.

Despite only BCT applied in the IG were identified, the provision of educational materials, pedometers or phone calls to maintain CG participants retention also could implicate the use of some BCT that were not being considered. Even in some studies [41], BCT were maintained during the follow-up period. Studies on inactive healthy adults have reported that an average of 5 BCT were applied in the CG leading to some change in PA behaviour in subjects who did not receive the intervention [46]. For this reason, future research should aim to identify the BCT used not only in the IG but also in the CG and during follow-up periods. This may explain why both groups experienced an improvement in PA levels, making it difficult to find significant differences between the groups. Additionally, analysing the BCT used in both groups could aid in understanding which techniques are crucial in generating increases in PA when behavioural improvement is observed in both the IG and CG.

### Limitations, strengths and future research lines

The primary limitation of this review is the small number of studies available on the effectiveness of combined PA and BCT interventions in promoting long-term PA behaviour among CRCS. The current evidence is reduced, with concerns regarding blinding, randomization and outcome measures. Further studies with higher methodological quality are required to gain a better understanding of the efficacy of these interventions. In addition, the clustering of PA measures collected at various points throughout the follow-up period in the meta-analysis may have introduced another potential source of limitation in the study. On the other hand, we were not able to compare baseline and follow-up outcomes with post-intervention ones in the meta-analysis because there was not enough data to do so. If we had been able to analyse this, we would have been able to see whether the improvements in PA up to post-intervention were maintained, increased or decreased compared to those achieved post-follow-up, and therefore, whether people were benefiting from optimal PA adherence or whether it was lost over time. For this reason, it is necessary to conduct more randomized controlled trials that not only examine improvements in PA after interventions but also investigate the maintenance of these changes over time. Furthermore, trials comparing PA interventions, BCT interventions and the combination of both are needed to determine whether long-term adherence to PA in this population is determined by the specific PA intervention, by BCT or by the need of both.

This SRM has also important strengths. Firstly, it is the first SRM to bring together evidence on PA and BCT interventions with a follow-up of 3–12 months in CRCS. Secondly, the SRM has a moderate certainty of evidence due to low inconsistency, indirectness and imprecision. Finally, the detailed analysis of the intervention and control conditions and the BCT implemented allowed us to identify possible key characteristics for PA adherence in CRCS.

## Conclusions

In conclusion, interventions that combine PA and BCT have been found to be effective in improving the maintenance of PA in CRCS after a follow-up period of 3–12 months. In order to identify, on the one hand, the possible minimum requirements to achieve increases in PA levels in the long term and, on the other hand, the discriminating characteristics between the most and least effective interventions, the common and differentiating characteristics of PA and BCT interventions have been extracted. In terms of PA, all interventions performed aerobic exercise and provided participants with pedometers, PA diaries and educational materials. However, among the included studies, the one with the largest effect size had distinctive features such as supervised exercise and facilitating access to fitness areas. Regarding applied BCT, all interventions were based on any TMBC and 12 common techniques have been identified. Nevertheless, comparing the studies according to their effect size, it was observed that the technique *demonstration of the behaviour* was only used in “very promising” or “promising” interventions and not in studies classified as “not promising”. In addition, *restructuring the physical environment* technique was exclusively applied in the intervention with the largest effect size. Thus, these techniques are suggested as possible discriminators between the most and least effective interventions. Further studies with higher methodological quality are needed to confirm these findings.

## Data Availability

The datasets generated and analysed during the current study are available from the corresponding author on reasonable request.
